# Extracellular vesicles as the next‐generation modulators of pharmacokinetics and pharmacodynamics of medications and their potential as adjuvant therapeutics

**DOI:** 10.1002/ctm2.70002

**Published:** 2024-08-21

**Authors:** Jiaqi Liu, Joel Z. Nordin, Andrew J. McLachlan, Wojciech Chrzanowski

**Affiliations:** ^1^ Sydney Pharmacy School Faculty of Medicine and Health University of Sydney Sydney Australia; ^2^ Division of Biomolecular and Cellular Medicine Division of Clinical Immunology Department of Laboratory Medicine Karolinska Institute Huddinge Sweden; ^3^ Division of Biomedical Engineering Department of Materials Science and Engineering Uppsala University Uppsala Australia

**Keywords:** context of use, emerging therapeutics, extracellular vesicle, nanomedicine, pharmacodynamics, pharmacokinetics, pharmacological modulation

## Abstract

**Background and main body:**

Pharmacokinetics (PK) and pharmacodynamics (PD) are central concepts to guide the dosage and administration of drug therapies and are essential to consider for both healthcare professionals and researchers in therapeutic planning and drug discovery. PK/PD properties of a drug significantly influence variability in response to treatment, including therapeutic failure or excessive medication‐related harm. Furthermore, suboptimal PK properties constitute a significant barrier to further development for some candidate treatments in drug discovery. This article describes how extracellular vesicles (EVs) affect different aspects of PK and PD of medications and their potential to modulate PK and PD properties to address problematic PK/PD profiles of drugs. We reviewed EVs' intrinsic effects on cell behaviours and medication responses. We also described how surface and cargo modifications can enhance EV functionalities and enable them as adjuvants to optimise the PK/PD profile of conventional medications. Furthermore, we demonstrated that various bioengineering strategies can be used to modify the properties of EVs, hence enhancing their potential to modulate PK and PD profile of medications.

**Conclusion:**

This review uncovers the critical role of EVs in PK and PD modulation and motivates further research and the development of assays to unfold EVs’ full potential in solving PK and PD‐related problems. However, while we have shown that EVs play a vital role in modulating PK and PD properties of medications, we postulated that it is essential to define the context of use when designing and utilising EVs in pharmaceutical and medical applications.

**Highlights:**

- Existing solutions for pharmacokinetics and pharmacodynamics modulation are limited.
- Extracellular vesicles can optimise pharmacokinetics as a drug delivery vehicle.
- Biogenesis and administration of extracellular vesicles can signal cell response.
- The pharmaceutical potential of extracellular vesicles can be enhanced by surface and cargo bioengineering.
- When using extracellular vesicles as modulators of pharmacokinetics and pharmacodynamics, the ‘context of use’ must be considered.

## Graphical abstract

Extracellular vesicles' potential in modulating medications' pharmacokinetics and pharmacodynamics.

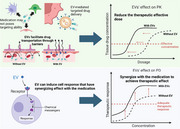

## 1 INTRODUCTION

### 1.1 Pharmacokinetics and pharmacodynamics

Pharmacokinetics (PK) and pharmacodynamics (PD) are two significant concepts for healthcare professionals and researchers that are critical to the design or study of the dosing and administration of drug therapy for different patient groups. PK describes the movement of drugs and metabolites in the body and consists of four aspects: absorption, distribution, metabolism and excretion. PD, on the other hand, refers to medications' biochemical and pharmacological effects at different in vivo concentrations, including their mode of action. In other words, PK is how the human body will affect the medications after they are administered, while PD is how the medications will exert their effects on the body.^1^, ^2^ A comprehensive understanding of the PK and PD of medications is essential for drug development, prescribing, administering, monitoring and management.

### 1.2 Extracellular vesicles

Extracellular vesicles (EVs) are lipid membrane‐surrounded structures secreted by cells to the extracellular space.^3^, ^4^ With a complex mixture of different combinations of protein, lipids and nucleic acid within the EVs’ cavity and on the surface of their membrane, EVs play a significant role in inter‐cellular communication. Moreover, it can affect the behaviour of recipient cells.^5^, ^6^ Furthermore, since the compositions of EVs are unique when they are released by specific cell types with particular communication purposes, EVs can be a diagnostic tool with high specificity, and they have great potential to be next‐generation therapeutic agents.^5^ EVs can be generated from multiple sources, including eukaryotic and prokaryotic cells. Till now the most studied EVs have been derived from bacteria, yeast, probiotics, plants, fungi and milk (including human milk), body fluids, immune cells, mesenchymal stem cells, cancer cells and cell lines.^7^ Many of these EVs have been shown to induce beneficial biological effects and proved their utility for various medical and pharmaceutical applications.^8^, ^9^ However, not all EV sources are suitable as parent cells to generate EVs for therapeutic purposes. For instance, EVs produced from cancer cells may potentially carry pro‐tumourigenic factors and increase the risk of tumour growth.^10^ However, these negative effects can be mitigated by genetic modification of the producer cells.^11^ Another source of EVs that may not be suitable for clinical applications is cells that undergo apoptosis because these cells produce particles containing damaged cell fragments and components, which become a part of the EV preparations.^10^ However, some EVs utilise the apoptotic component to exert their therapeutic effects, for instance, Mesenchymal stem cells (MSC)‐derived EVs provide anti‐inflammatory effects by their apoptotic bodies.^12^ EVs may carry a wide range of allogenic proteins that may induce or inhibit immune response, hence potentially introducing resistance to certain pathogens. For instance, cancer‐associated fibroblasts under anti‐cancer therapy may generate EVs that induce phenotypic impact on recipient cells, resulting in EV‐mediated transfer of drug resistance.^13^

Since EVs have the structure of membrane vesicles, which has potential application for drug encapsulation and their surface structure can be modified by EV engineering, EVs can be developed as effective drug delivery vehicles for medication with suboptimal PK/PD profile.^14^, ^15^, ^16^ There is evidence that PK/PD is influenced by the secretion and exchange of paracrine signals by cells via miRNA carrying EVs.^5^ The exact mechanism of action remains uncertain since there is lack of in vivo experiment data; EVs are vital for this communication, since miRNA requires protection from serum enzymes, or it will be rapidly degraded.^5^, ^6^ Second, EVs can interact with recipient cells by the EV surface and EV surface proteome that contain a plethora of receptors and ligands from the source cell, and thus the EV can be seen as a signalosome capable of both surface signalling as well as cytoplasmic and nuclear signalling through delivery of signalling molecules, such as proteins. In summary, EVs are recognised as crucial mediators of cell‐to‐cell communication and/or crosstalk between organs with the potential to modify the PK and PD properties of medications as both drug vehicles and drug uptake modulators. With EVs’ ability to alter the behaviour of recipient cells (e.g., sensitise cells to modulate a drug uptake) and their capability to deliver drugs that have poor bioavailability directly to specific cells, they are believed to have considerable potential in optimising drug therapies that are limited by poor PK/PD profile of the drugs, as both a modulator and a delivery agent.

## 2 THE URGENT NEED FOR A MODULATOR OR A VEHICLE FOR MEDICATIONS

### 2.1 The underlying challenges in drug discovery and clinical settings that are caused by PK and PD‐related issues

**FIGURE 1:**
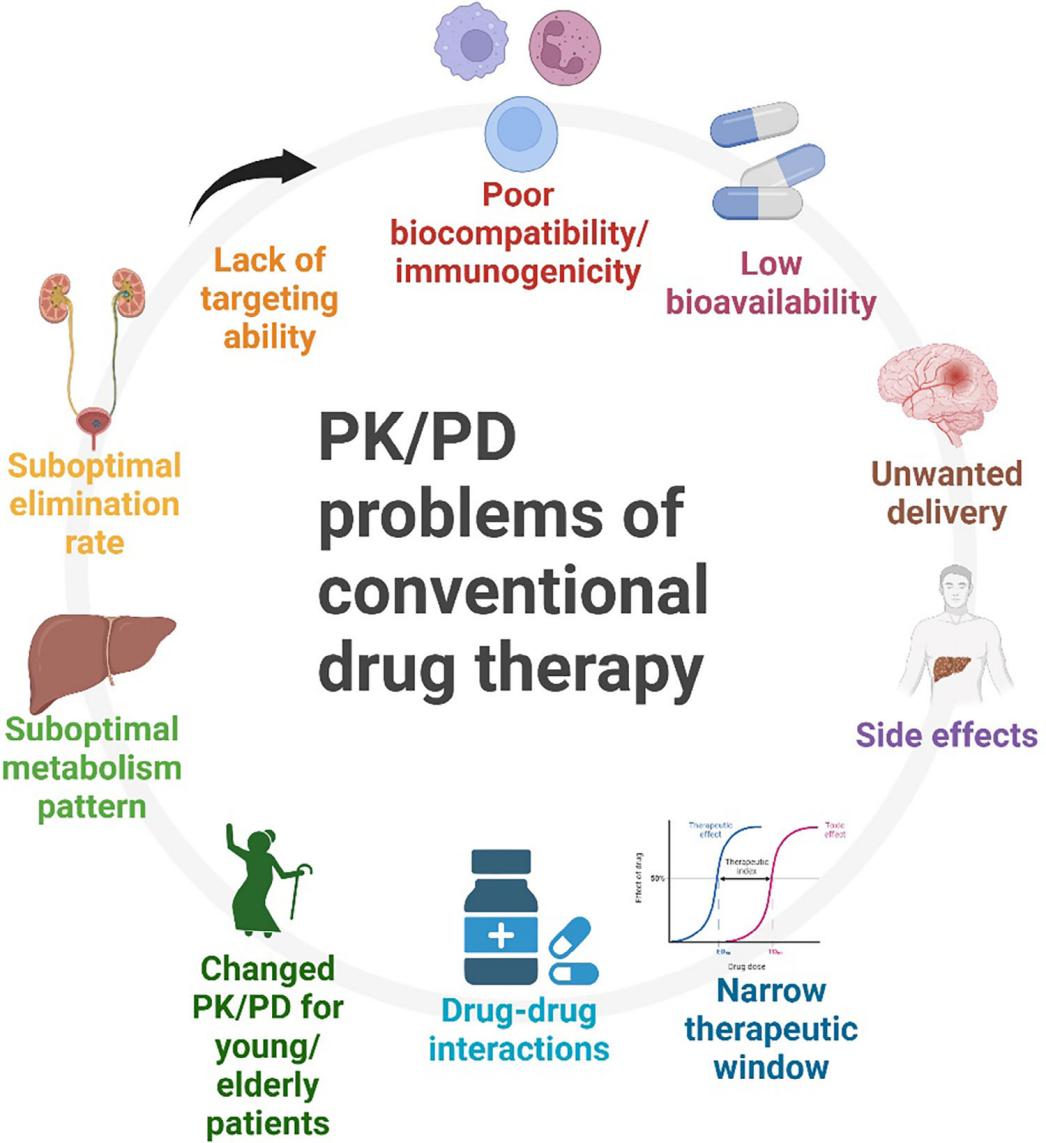
Common pharmacokinetics/pharmacodynamics (PK/PD)‐related issues in conventional drug therapy that create challenges for drug development.

In an ideal situation, a ‘perfect’ medication will have the most desirable PK and PD profile, meaning it will only move to the target tissue or organ without exerting any unwanted effects. However, this is not the case in the real‐world situation. Medications may have undesirable PK and PD characteristics, which means they may not have an ideal bioavailability; drugs may also move to untargeted tissues/organs; our body may eliminate the drugs too fast or too slow; and they may cause unwanted or harmful effects to healthy tissues/organs. Moreover, patients’ health conditions and existing therapy plans will further influence the PK/PD and complicate the choice of medications and their dosage. This is one of the leading causes of polypharmacy (using five or more medications concurrently), and polypharmacy, which is typically caused by the unnecessary addition of drugs in the therapeutic plan due to problem or drug‒drug interaction of existing medications in the plan, is becoming an emerging problem of the healthcare system. Polypharmacy will additionally complicate healthcare professionals’ therapeutic planning since it is almost impossible to predict how a combination of more than four drugs will affect the PK and PD of each drug.^17^ Moreover, some medications may affect the PK/PD of several drug classes, which may limit the choice of medication during prescription since certain combination of drugs are usually prohibited. Thus, medications that have problematic PK or PD issues may put healthcare professionals into a dilemma when prescribing, monitoring and managing the therapeutic plan (Figure 1).

It is inevitable for healthcare professionals to prescribe medications that have a narrow therapeutic index (NTI) to patients with certain health conditions. Medications such as chemotherapy, warfarin, phenytoin, and digoxin are examples of commonly prescribed NTI medications. Their effective and toxic doses are very close, making it much more difficult for dose management and much more costly for therapeutic monitoring.^18^ Moreover, although some medications may be well tolerated and have a broader therapeutic index, their in vivo concentration may change dramatically due to PK drug‒drug interactions. Some commonly prescribed medications may have a noteworthy impact on the absorption, distribution or elimination of other medications, which may cause severe toxicity. Drugs such as H2 antagonists and proton pump inhibitors (PPI) can alter the pH of the gastrointestinal tract and affect the absorption rate or place of absorption for medications such as β‐blockers. As for distribution, an example could be the co‐administration of warfarin and diclofenac, which bind competitively with albumin. Therefore, administration of one of the medications will increase the concentration of the other free drug. Metabolism and excretion of target medications can be affected by other medications as well, the addition of CYP enzyme inducer or inhibitor medications significantly affects the metabolism of different medications in patients’ therapeutic plans. Additionally, inducers or inhibitors of transmembrane proteins such as P‐glycoprotein can affect the excretion of specific drug classes, such as antineoplastic drugs, calcium channel blockers and digoxin.^19^ Studies have also shown evidence that medications may interact at the PD level.^20^ In these situations, a modulator of PK/PD or a delivery system would be extremely helpful, and they may enable the concurrent use of some medications that are previously considered to cause PK/PD interactions.

Another challenge for drug development is that medications that have desirable PK and PD profiles are infrequently discovered or developed, which may introduce limitations and risks in their usage. Take conventional chemotherapies as an example; studies have suggested that their therapeutic effects are achieved by increasing the dose administered, which will cause a large proportion of cytotoxic medications to be absorbed by healthy tissues or organs.^21^ The mechanism of action of chemotherapy is to damage and kill the cells. However, they do not possess targeting ability, which means healthy cells are also exposed to their cytotoxic effects. Consequently, since the absorption and distribution of chemotherapy lack specificity, only a tiny proportion of chemotherapy will reach the diseased tissue, which may not reach the therapeutic concentration to kill the cancer cells due to its problematic PK profile. On the flip side, if prescribers keep increasing the dose to achieve a therapeutic effect and neglect the systematic absorption since healthy cells do not have an adequate protective mechanism against chemotherapies, patients may experience serious adverse effects, which will have a substantial negative impact on patient's quality of life and adherence to the therapy. Thus, healthcare professionals will have to put much more effort into managing or monitoring medications such as chemotherapy and immunomodulators. There are many potential solutions for the dilemma; a delivery system can be developed to reduce systemic adsorption, hence lowering the risk of adverse effects. Moreover, a much‐anticipated method is to develop an adjuvant therapy that can act as a pre‐treatment or co‐treatment for the patient to modulate the PK of those medications that can facilitate the absorption and distribution of medications to the diseased tissue or organ while exerting a protective effect for healthy cells against the medications. In other words, the new adjuvant therapy will optimise the PK profile of those medications to give them specificity to targeted tissue or organs.

Patients’ health conditions also have an impact on the PK and PD of medications and make it even more difficult for therapeutic management. Studies have illustrated that pre‐existing diseases can affect PK and PD; a typical case would be a patient with chronic liver disease since the patient will have impaired drug uptake and oxygen limitation.^22^ Geriatric patients are one of the patient groups that have a considerably high risk of having PK and PD‐related problems. The geriatric group has high inter‐individual differences in organ function and homeostasis; additionally, their drug clearance and elimination may also be reduced with ageing. Furthermore, ageing will also alter their sensitivity to medications.^23^ Studies also suggest geriatric patients have an increased prevalence of polypharmacy and comorbidities, which create more problems for healthcare professionals in managing their treatment plans. Paediatric patients are another patient group with significantly altered PK and PD; studies demonstrated that their PK will change dramatically throughout the first 5 months of their lives.^24^ Moreover, both groups are highly vulnerable to adverse reactions compared to adults, which requires regular monitoring and management of their medication plan. Unfortunately, due to ethical issues, there is a lack of clinical trials for those patient groups. Hence, there are insufficient dose instructions in therapeutic guidelines for those groups, which puts prescribers into a dilemma and limits the available choices of medications since they have to find the balance between efficacy and safety in certain situations. Furthermore, studies have also shown that genetic polymorphism also has a significant impact on the PK and PD of different medications.^25^, ^26^, ^27^ Developing a new adjuvant therapy that can modulate PK and PD may break the stalemate for prescribers since it can make therapeutic management more flexible.

### 2.2 Existing solutions for PK/PD‐related issues and their limitations

The existing methodologies to solve PK/PD‐related problems all have their limitations and may not be suitable for all patient groups. Thus, there is a real need for new strategies that can help optimise therapeutic plans, especially for drug classes such as antibiotics, sedatives, analgesics, chemotherapies and immunomodulators. One of the ideal strategies is individualised dose optimisation plus drug scheduling; this is more common in hospital settings and intensive care, especially for particular patient groups (such as older people, children and pregnant women).^14^, ^15^ However, this method requires regular patient monitoring and communication, which create an extra workload for healthcare professionals and be more costly for patients. Moreover, it would be even more challenging to achieve in community settings. Another method is changing the route of administration; a typical example is changing an oral formulation to an intravascular injection to bypass the first‐pass metabolism. Sometimes, infusions are preferable if sustained release is required.^1^ However, injections or infusions usually require skilled professionals, which is less convenient for patients and may affect their adherence; additionally, not all medications are suitable for changing routes of administration; for example, suspensions or oily solutions cannot be injected. Moreover, adjuvant drugs are developed to improve conventional therapies; for example, nivolumab, a medication that can stimulate immune function, was recently approved by the FDA to treat lung cancer alongside conventional chemotherapy to enhance the overall therapeutic outcomes.^17^ However, choosing effective adjuvant therapies is still extremely limited.^14^ Another example of classical PK/PD modulator application is using P‐glycoprotein inhibitors to reverse multidrug resistance caused by overexpression of ATP‐binding cassette transporter P‐glycoprotein. Three generations of p‐glycoprotein inhibitors, such as verapamil, dexniguldipine and tariquidar, have been developed and examined in preclinical and clinical trials. However, they all demonstrated poor outcomes in improving the therapeutic efficacy of conventional cytotoxic drugs due to problems such as poor affinity to P‐glycoprotein, concerning adverse effects and difficulty in dosage management, thus a new generation of P‐glycoprotein modulator is highly anticipated.^17^, ^19^ Additionally, even though we have some adjuvant therapies that have shown to be successful (such as using immunomodulators to enhance chemotherapy efficacy in cancer treatment, pharmacogenetic modulation, circadian administration and drug scheduling), healthcare professionals still need to be extremely careful when adding a new medication to patients’ therapeutic plan; moreover, strict drug scheduling may not be suitable for all patient groups since it may cause adherence issues.^15^ In many circumstances, doctors or patients may add a new drug to treat the side effects of a previously prescribed medication, which may cause polypharmacy and potentially introduce new drug‒drug interactions. Thus, a safe and target‐specific delivery vehicle is much anticipated, and healthcare professionals are still looking for the development of more adjuvant modulators (Table 1).

**TABLE 1:** Summary of the existing solutions for pharmacokinetics/pharmacodynamics (PK/PD) problems.

Existing solutions for PK/PD problems	Limitations
Individualised dose adjustments and drug scheduling	Costly
	Time consuming
	Usually not available for all patients
Changing the route of administration	Not suitable for all medications/patient group
	Create extra healthcare burden
	Some routes require extra clinical skills
Add another conventional medication	Increase risk of toxicity
	May introduce drug‒drug interactions
	May cause polypharmacy

## 3 PHARMACOKINETICS AND PHARMACODYNAMICS OF EVs

**FIGURE 2:**
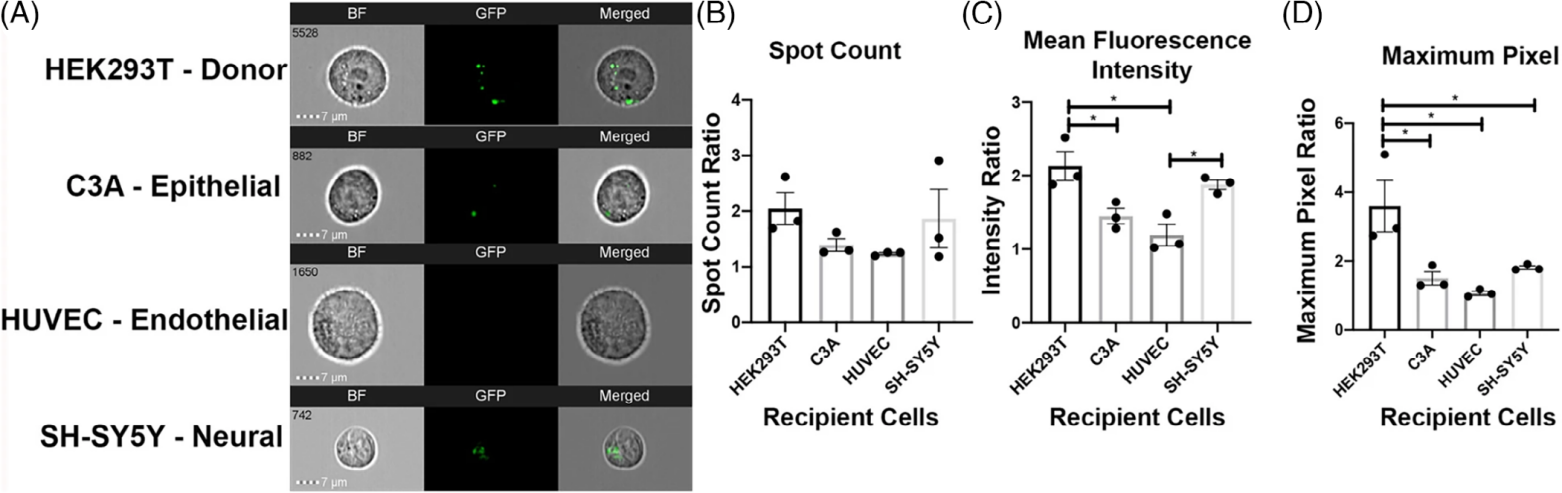
Extracellular vesicles (EVs) internalisation assays. (A) Imaging flow cytometry (IFC) image shows the internalisation of HEK293 EVs, which are stained with green fluorescence. (B‒D) Quantification assays of the internalisation of HEK293 EVs indicate that EVs produced from HEK293T cells display a higher uptake ratio to their donor cell (HEK293T) compared to other cell types.^30^

Although EVs have great potential as drug delivery systems and therapeutic agents, their PK/PD profiles are still not fully established. Since the concept of using EVs as drug vehicles and next‐generation therapeutics is relatively new, a majority of PK studies are done in animal models instead of human models; however, they still show EVs tend to concentrate in diseased organs, which shows their potential of possessing specificity to target tissue/organ;^28^ however, the majority of exogenously injected EVs are taken up by macrophages in liver and spleen. The biodistribution of EVs can be affected by many factors, including the dose, route of administration, their cell origin and intrinsic or engineered targeting properties.^29^ Study has shown the bio‐distribution of EVs produced by HEK293 cells in different organs with different dosages, which illustrates that at the lowest dosage, the majority of the EVs were distributed to the liver while there was increased distribution to other organs as the dose increased.^29^ The study also demonstrate different patterns of biodistribution with different routes of administration, which suggests that the biodistribution of EV can be modulated by changing the route of administration, intraperitoneal injection and subcutaneous injection have significantly lower accumulation in the liver (35% and 30%, respectively) and spleen (5% and 2%, respectively) compared to intravenous injection (which has 60% accumulation in liver and 12% accumulation in spleen) but increased distribution to other organs.^22^ Furthermore, by measuring the total tissue fluorescence, they found that different routes of administration also result in different overall body absorption of EVs. Another study also discovered that EVs that are produced from HEK293T cells display a higher uptake ratio to their donor cell (HEK293T) compared to other cell types, a detailed uptake preferences difference is illustrated in Figure 2, which used three different methods (spot count, mean fluorescence intensity and maximum pixel) to test out the amount of EVs, which are produced by HEK293T absorbed by different cells, they all suggest that the EVs are preferably absorbed by their donor cells.^30^ Studies indicate that EVs have various theoretically advantageous PK/PD profiles compared to other existing drug delivery systems.^31^ It is important to note that these studies suggested that these targeted EVs increased the proportion of drugs reaching a specific tissue or organ; however, they did not prove the exclusive and absolute targeting of specific cells or organs. Since EVs have a similar membranous structure to the membrane of human cells, it is believed that they can penetrate barriers in the human body, such as the blood‒brain barrier, epithelium and cell membrane.^28^, ^31^ However, there is lack of in vivo experiments in human that support EV's capability in penetrating central nervous system, EVs demonstrate limited capability to reach distal tissues (e.g., brain) due to extensive accumulation in liver. Thus, the effectiveness of EV's barrier penetrating property still requires more evidence.

Stability and biocompatibility are also considered to be two significant theoretical advantages of EVs. Stability in blood circulation is essential for EVs and their cargo to reach the target organ. Since EVs are scavenged by monocyte, macrophage or reticuloendothelial system, the EVs that are not surface engineered have rapid clearance in bloodstream, which could be an obstacle for their clinical application. Surface engineering of EVs have been developed to solve this problem, EVs demonstrate increased circulation time after surface engineering with albumin binding domains.^32^ The engineered EVs exert robust binding capacity to human serum albumins (in vitro) and mouse serum albumins (after injection in mice), which demonstrated promising increase in circulating time and much higher accumulation of EVs in lymph node and solid tumour.^32^ Since a large proportion of EVs are produced from normal human cells, they also demonstrate less risk of causing harm to the patients because they are more compatible with human tissue/cells than standard formulations.^31^ EVs’ membranous structure also gives them the potential to be loaded with a ‘cocktail’ of cargo.^33^ Moreover, since we can engineer the surface structure during production, EVs’ PK/PD profile is more customizable than conventional medications.^31^

In addition to EVs’ customizable PK/PD profile, EVs can be delivered with multiple routes of administration, which is essential for modulating targeted delivery and biodistribution of the EVs. With the variety of routes of administration, healthcare professionals will have more options for different therapeutic scenarios. Intravenous injection is the most common route of administration of EVs, it provides superior biodistribution and therapeutic efficiency, but it also has the highest rate of clearance, which may reduce the duration of the therapeutic effect.^34^ Moreover, EVs can be administered orally, it has promising bioavailability and it has lower toxicity compared to free active drugs.^35^ Orally administered EVs also demonstrate superiority in providing targeted and localised treatment for disease in the gastrointestinal tract.^34^ Furthermore, EVs demonstrate great in translocating to the brain when they are delivered intranasally, although this route may have a lower therapeutic effect due to short retention time.^36^ In addition, inhalation of mesenchymal stem cell‐derived EVs demonstrated non‐invasive and targeted therapeutic effects in treating COVID‐19‐induced lung inflammation and acute lung injury.^37^ EVs can also be applied via topical and transdermal administration. A study has found that curcumin, a drug for skin inflammation, demonstrated improved cellular uptake stability when it is encapsulated in EVs via dissolvable microneedle arrays.^38^

There are also several biological barriers for EVs to overcome as drug vehicles or therapeutic agent. To have sufficient therapeutic effectiveness, EVs need to avoid rapid immune clearance, which can be achieved by modifying the EVs with surface structures (such as CD47 and PEGylation) that protect EVs from clearance and increase the duration of therapeutic effects.^39^ Another barrier for EVs to transvers is the plasma membrane, which plays an essential role in regulating cellular uptake of macromolecules that are too large and/or charged to transverse the membrane by diffusion. EVs can be internalised through a combination of pathways, including endocytosis, micropinocytosis, phagocytosis and direct fusion.^40^ However, the exact unloading process of EV cargo is still unclear, some in vivo studies demonstrate that EVs accumulate in the endo‐lysosomal compartment, but it is uncertain how EVs can protect their cargo from lysosome‐mediated destruction.^41^ This EV uptake mechanism give some insight of possible EVs engineering to facilitate more effective EVs or cargo uptake. Virus and bacteria are capable to escape the endosome before lysosome destruction; thus, surface‐engineering EVs with bacterial or viral protein might be a viable method to enhance the cellular uptake of EVs and their cargo.^41^

EVs that have therapeutic potentials can be isolated from different sources, apart from EVs produced by human cells, plant and microorganisms such as bacteria and microalgae are also considered to be promising natural biosources of EVs.^42^, ^43^ Moreover, EVs from different sources exhibit different properties and unique effects when they are administered. Recent preliminary studies demonstrated that plant‐derived EVs can exhibit multiple therapeutic effects, including restoring gut homeostasis and triggering specific immunomodulatory effects. These EVs can be isolated from the juice, root and the seeds of the plants (such as grape, broccoli and ginger), and they demonstrated outstanding stability in gut environment.^44^ Plant‐derived EVs also exhibit promising biocompatibility in the human body since they are less likely to triggering inflammation response or tissue damage when comparing them with conventional liposomal products.^42^ EVs that are isolated from humans’ intestinal microbiota is a modulator of homeostasis and bacterial colonisation in gut. Moreover, bacteria‐derived EVs demonstrate diverse beneficial or pathological effect on the gut microbiota when they are isolated from different bacteria due to the differences in their intrinsic cargo composition (such as protein, DNA, RNA and the EV metabolites).^42^, ^45^ Furthermore, microalgae are also considered to be a cost‐effective source of EVs: they are currently under preclinical development as a promising drug delivery system since they exhibit great potential in overcoming mammalian natural barriers (e.g., blood‒brain barrier and gut barrier) while transporting the loaded therapeutic cargo to specific tissues.^45^ Therefore, EVs can exhibit unique PK and PD properties when they have different source of biogenesis; The diversity of EVs sources enables experts to have diverse options of EV subtypes fulfil the PK/PD requirements of different therapeutic scenarios.

## 4 EVs AND PHARMACOLOGICAL MODULATION

### 4.1 Theories that support links between EVs and medications’ PK/PD profiles at the cellular level

EVs play essential roles in cell signaling, which can cause changes in protein synthesis, receptor activities and behaviours (such as inflammation and immune response).^46^ Since EVs carry a range of bioactive molecules (e.g., protein/peptides), surface receptors and genetic molecules (such as protein‐coding mRNAs and regulatory miRNAs), they show potential in the pre‐setting cellular environment to regulate medications PK/PD profile.^47^ When EVs are engulfed by the recipient cells via endocytosis, EVs’ membrane constituents will be delivered to the cell membrane; moreover, a range of functional and/or genetic cargo will be delivered to the cytoplasm and cell nucleus, which can potentially cause functional and behavioural changes in the recipient cells.^46^, ^47^ The protein‐coding mRNAs can facilitate the synthesis of enzymes or protein molecules that competitively block specific receptors/transporters. Moreover, the regulatory miRNAs can be delivered to the cell nucleus to regulate the expression of particular genes, which can up/downregulate the number of specific enzymes or receptors.^48^ This mechanism can potentially be utilised to achieve regulation of PK/PD for different medications via different EV modifications.

It has been found that medication intake plays a significant role in the biogenesis of EV.^49^ Calcium level is one of the critical modulators of EV biogenesis, drugs that can trigger interference of calcium homeostasis are anticipated to impact the biogenesis of EVs. A study has shown that amiloride, which is a calcium channel inhibitor, has a suppressive effect on the fundamental and stimulated biogenesis of EV.^50^ Moreover, calcium channel blockers and β‐blockers also showed a reduction effect on the activation of endothelial cell activation, which can lead to the reduction of EV uptake, resulting in an increased EV population.^51^ This mechanism can explain some drug‒drug interactions of certain medications from a PK perspective since EVs are potential vehicles of medication that will facilitate intercellular transportation of drugs to the next cell and enhance the distribution of medication in local tissue and even the entire body. Thus, one of the possible mechanisms is that when the medication that can facilitate EV biosynthesis contacts the cell membrane/cytosol, it will trigger the production of certain EVs, which enhance the intercellular transportation of certain medicines. Conversely, if we introduce a medication that can antagonise the biosynthesis of EV, we can downregulate the distribution of drugs.

EVs also demonstrate an association with the PD of medications. Diseases can also trigger the biogenesis of EVs, which plays a crucial role in disease pathogenesis, cell recovery and tissue regeneration.^49^, ^52^ It has been found that EV biogenesis is associated with the pathology and or recovery of many diseases, including diabetes, multiple cardiovascular diseases (including acute thrombosis, myocardial infarction, hypertension and angina) and atherosclerosis.^49^ The biogenesis of EVs can alter the progress of disease and recovery in multiple modes of actions: since EVs have an intrinsic signalling function, the symptoms and/or damage of some diseases (e.g., thrombosis and acute myocardial damage) are caused by the biogenesis of EVs. Increased EV production may also be caused by the homeostatic response, either directly due to homeostatic disturbance that caused by the disease or sent out by our body to signal the restoration of normal homeostasis (e.g., the gut microbiota that produces outer membrane vesicles that could impact tissue homeostasis, promoting immune response and enhance clearance of toxins created by certain bacteria).^49^, ^53^ It may also be triggered by our body to produce EVs to signal/induce wound healing and tissue regeneration (e.g., bone‐derived EV signals the communication between osteoblast and osteoclast, which modulate osteogenesis).^52^ Thus, depending on the mode of action, EVs can affect patients’ bodies in both beneficial and harmful ways. If we utilise the EVs that can contribute to the recovery of certain diseases, their beneficial effect can synergise with conventional medications and improve the PD profile of drugs. By having a better understanding of EVs’ role in disease and therapy, it is anticipated that EVs can be utilised to understand and modulate the therapeutic effects and modes of action of medications and, hence, modulate their PD profile.

### 4.2 EVs as modulators of PK

For medications that have relatively good PD profiles but with suboptimal PK, EVs can be utilised as a drug delivery system to avoid the intrinsic PK profile of those drugs. Over the past few decades, scientists’ interest in EVs has grown rapidly since they possess many theoretical advantages as drug vehicles.^14^ The membrane vesicle structure is suitable for loading cargo and remains with a stable structure; moreover, unlike conventional drug delivery systems, which generally only deliver one type of medication, EVs can carry different categories of medications (such as peptides, proteins and RNAs) at the same time.^33^ For drugs that have poor absorption or poor distribution, since EVs enclose them within the cavity, their intrinsic physiochemical properties are masked by the EVs. Additionally, they are protected against early metabolism. Previous study has found that, under RNase treatment, the degradation of miRNAs is substantially reduced after they are loaded in fruit‐derived EVs, which increases the survival rate of miRNA by over 50%.^54^

Drugs can be loaded into EVs endogenously (during EV biogenesis) or exogenously (after isolation of EVs) via active or passive loading strategies. These strategies include genetic modifications of producer/donor cells, and molecular, physical and chemical approaches to load a drug either during the biogenesis of EVs or after the EVs were produced and isolated. Loading the drug can be achieved by either attaching drug molecules to the EV surface (add‐on), typically via chemical linkers, or enriching internal EV cargo (add‐in) using both active and passive strategies.^55^, ^56^ Active strategies rely on disruption of EVs’ membrane to allow cargo loading, while passive strategies utilise diffusion with no membrane disruption.^55^

The selection of the loading method depends on multiple parameters, such as the nature of the cargo (cargo's molecular size, concentration, aggregation and polarity) and the nature of the EVs (their parent cell, isolation method, loading capacity and loading efficiency). Passive loading is a relatively simple method for loading drugs into/onto EVs. The drug loading can be achieved by co‐incubating the parent cells or isolated EVs with the molecules of choice. This process facilitates the encapsulation of the drug into EVs during biogenesis, and either the translocation of drug molecules through the EVs’ lipid membrane or their attachment to the surface. The attachment to the surface occurs mostly via electrostatic interaction. Such passive loading is easier to conduct, and it does not require specialised equipment to achieve desirable loading efficiency. However, this strategy is more suitable for small‐molecule drugs (e.g., curcumin) since large synthetic molecules are less likely to be transported to the cavity of EVs. At the same time, small molecules are more susceptible to diffusing out of EVs due to their smaller molecule size, hence the possibility to store them for a longer time in buffers might be limited.^55^, ^56^

To enable more effective loading of drugs of different types, membrane permeabilisation methods that utilise natural detergents such as saponin can be used. These methods ‘open’ up the membrane which facilitates the passive flow of drugs into EVs.^57^ This loading strategy enables higher loading efficiency compared to passive loading without permeabilisation, while it remains relatively simple and cost‐effective. However, the residual detergent and unused drug must be removed using extensive washing techniques. The washing will inevitably result in the EV loss. In addition, it is not clear whether detergents would disturb EV membrane microdomains, which are essential for biodistribution and cellular uptake, hence further investigation is required.^55^, ^56^

Sonication is another loading technology that induces temporary mechanical disruption to the EV lipid membrane—sonoporation. Ultrasound dilates the membrane of EVs which enables more effective transfer of the drug into EV. This method provides high loading efficiency for a wider range of cargos regardless of their physical and chemical properties compared to other technologies. However, due to the heterogeneity of EVs and differences in their subpopulations, it is not trivial to achieve uniform loading and there is a risk of damaging some of the EVs due to the relatively high energy of ultrasound. Currently, there have not been systematic studies that determined the correlation between EVs (the size, structure and composition), the ultrasound parameters (intensity, amplitude and time) and the membrane dilation (sonoporation, drug loading ability). As a result, the ultrasound‐enabled loading protocols have not been standardised. Furthermore, sonication may not be viable for some cargos (e.g., plasmid DNA) since it may lead to cargo aggregation or degradation and hinder their therapeutic effects.^55^, ^56^

Electroporation can also create temporary pores in the EV membrane to make them more permeable for cargo loading, this is achieved by the application of an electric field to the EVs. The loading efficiency of this technology may vary depending on the properties of the EVs and the cargo. Similar to sonoporation, this technology still requires further studies since there is a lack of standardised electric field parameters that can provide consistent cargo loading and it is not fully understood whether electroporation can affect the integrity and/or functionality of the EVs.^55^, ^58^

Besides strategies that enable loading the drug molecules into EVs, some strategies link the drug to the EV surface intraluminally during the EV biogenesis. One such advanced strategy uses Tandem MS2‐coated proteins (MCP) to link EV surface protein (CD63) and an aptamer (MS2 aptamer) of the Cas9 ribonucleoprotein complex (Cas9 RNP), which loads Cas9RNPs on the surface of KEK293T‐derived EVs.^59^ While effective, this strategy results in a substantial retention of the cargo on the EVs’ membrane, which hampers cargo release from the EVs to the recipient cells. To overcome this issue, a cleavable linker can be engineered to enable ‘on‐demand’ and effective cargo release. In line with such approach, Elsharkasy et al. incorporated a photocleavable domain into the linker (Tandem MCP). Upon exposure of these EVs to 400 nm UV light, the domain is cleaved, resulting in cargo release from the EVs’ surface.^59^ Such systems enable external activation of the cargo release, primarily occurring in the areas exposed to the trigger (UV light in this case). This approach mitigates the risk of off‐targeted cargo delivery (Table 2).

**TABLE 2:** Advantages of extracellular vesicles (EVs) as a therapeutic agent and drug delivery vehicle.

EVs’ potential therapeutic roles	Advantages
Intrinsic therapeutic potentials	EVs’ structure is suitable for cargo loading Better biocompatibility and immunogenicity profile Intrinsic targeting ability
Drug delivery vehicle	Can help cargo bypass immunobiological barriers and pass through structural barriers Can be engineered to increase specificity or targeting ability to specific cells
A modulator of pharmacokinetics/pharmacodynamics	EVs play an essential role in cell communication and cell functions such as absorption and metabolism It can provide intrinsic or engineered therapeutic effects that synergise with other medications

Additionally, EVs demonstrate potential to provide sustained drug release with sufficient surface engineering. Although there are limited studies regarding this subject, we can anticipate their potential with the success of liposome, which is another drug delivery system that has a similar structure with EVs. Liposomes are also spherical‐shaped lipid bilayer vesicles that can be utilised to encapsulate drugs as drug delivery vehicles in their core cavities. The difference between EVs and liposomes is that EVs are naturally secreted by the cells with variable inherent lipid, protein and nucleic acid based on their cell origin, while liposomes are artificial vesicles without protein and nucleic acid in their composition, and they have a slightly different lipid composition.^60^, ^61^ Without surface engineering, both EVs and liposomes have substantially diminished bioavailability within the blood circulation due to the formation of protein corona and rapid clearance.^60^, ^62^ Polyethylene glycol (PEG) is widely studied as a surface coating to extend the blood circulating duration of nanoparticles.^62^ Moreover, both EVs and liposomes can be coated with PEG.^62^ Studies have shown that PEG encapsulated liposomes can achieve a much more stable drug concentration for patients. For example, cisplatin, which is a chemotherapy, is typically administered via intravenous infusion, and there will be only 10% of the medication remaining in the plasma 48 h after the infusion; however, SPI‐077, cisplatin encapsulated PEG‐coated liposomes, can achieve a much more stable concentration, with nearly no change in the first 72 h and the concentration is expected to be stable for more than 10 days.^62^, ^63^ Due to the similarity in structure, it can be anticipated that similar strategies can be used for EVs to modify their circulation duration, and hence, their PK. This finding is essential for cancer treatment since it can potentially lower the therapeutic effective dose of chemotherapy. Chemotherapies have a high risk of causing substantial damage to healthy tissues/organs due to the cytotoxicity property of the drug, healthcare professionals are required to prescribe higher doses of medication to achieve desirable therapeutic effects since a large quantity of the medication is delivered to off‐target cells/organs or removed by the body. Having EVs as highly specific and stable drug vehicles can potentially lower the initial dose for those therapies, thus reducing the risk of causing harm to patients. Moreover, having a more stable plasma concentration also means there will be an extended therapeutic effect since drug concentration in the patient's body will be within the therapeutic range for a more extended period of time, which can reduce the frequency of chemotherapy administrations and reduce the overall quantity of chemotherapies administered to patients, hence, reduce the risk of liver and kidney problems that potentially caused by the treatment.

It is essential for a therapeutic agent to be able to access and accumulate in the target tissue and release specific biological signal or be taken up by the target cells. Many drug classes have difficulties reaching their target organs since there are immunological and structural barriers present. EVs from different cell origins demonstrated their capability to cross the blood‒brain barrier, epithelial barrier and local cell membrane, which is essential for facilitating the medications to overcome those barriers; an example of this application is using EVs to deliver anti‐inflammatory drugs, which cannot cross the blood‒brain barrier, to access the central nervous system.^14^, ^63^, ^64^ Studies also found that EVs have intrinsic cell targeting properties, which means it has excellent potential to make the treatment more specific to the diseased cells and reduce unwanted systemic distribution.^65^, ^66^

Various features of EVs, with surface and corona composition being particularly important, lead to their preferential targeting of specific cell types. Key functional elements for this targeting include naturally expressed proteins such as tetraspanins and proteoglycans (e.g., glypican) presented on the EV surface.^67^ Furthermore, the presence of integrin cell adhesion guides EVs to selectively bind to different targets, enhancing their effective delivery to specific tissues such as the brain (via ITGβ3), liver (via ITGαvβ5) or lungs (via ITGα6β4 and ITGα6β1).^68^ The composition of the EV lipid bilayer is another factor that can alter the targeting ability of EVs and their internalisation by cells. For instance, the presence of phosphatidylserine within the lipid structure can induce a higher uptake in macrophages.^69^ Moreover, EV surface glycans that are present on the EV surface affect the targeting by interacting with the surface receptors of cells. Glycans on mesenchymal stem cell‐derived EVs have been shown to interact with lectin receptors of HeLa cells, which facilitate the recognition and uptake of these EVs.^70^

It is worth noting that changes to the composition of the EV surface including the presence of different coronas (both soft and hard coronas) that are dependent on the microenvironment in which EVs were both secreted and are present alter the surface charge of EVs. Since charge‐dependent electrostatic interactions of any nanoparticle with cells can facilitate cellular targeting and uptake, the surface charge of EVs is also considered as a factor to target as well as up‐ or downregulate the uptake of EVs by different cell types. For example, Matsumoto et al. showed that EVs have reduced targeting to murine macrophages when they are negatively charged compared to their neutral form.^71^ Another surface‐engineering method to enhance targeting is conjugating EVs with peptides that can be recognised by receptors that are solely or predominantly expressed on specific cells. For instance, the conjugation of EVs with neuropilin‐1‐receptor‐targeted peptide increased targeting to glioma cells due to the high expression of neuropilin‐1 receptors on this cell type.^72^ These studies showed an increased overall drug dose delivered via EVs. However, direct evidence of improved EV targeting was not presented. The drug's increased presence could result from either enhanced targeting or improved EV internalisation and drug unloading within cells.

**FIGURE 3:**
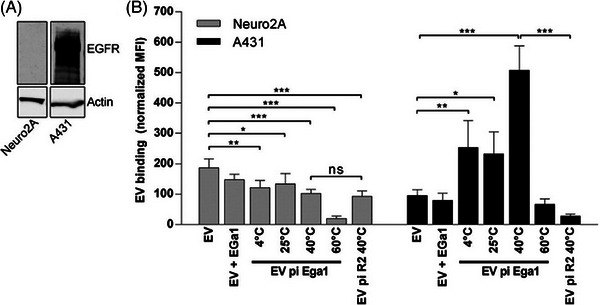
Post‐insertion (surface engineering) of PEG‐Ega1 and PEG‐R2 nanobodies demonstrate different effects on extracellular vesicles’ (EVs’) binding affinity. (A) Western blot analysis of EGFR expression of two different cell types, Neuro2A and A431, demonstrates specificity to A431 after PEGylation. (B) EVs surface engineered with PEG‐Ega1 nanobody (EV pi Ega1) have decreased binding to Neuro2A cells compared to non‐engineered EVs. However, they have increased binding to A431 cells compared to non‐engineered EVs. If we change the surface‐engineered nanobody to REG‐R2 (EV pi R2). In that case, the change of effect on EV binding is completely different, which slightly decreases binding to Neuro2A and dramatically decreases binding to A431.^74^

These feature is especially significant for medications with NTIs, medicines that may cause severe adverse reactions, and medications whose mode of action is damaging or killing cells. Therefore, EV as the delivery system can lower the therapeutic effective dose, avoid unwanted systemic effects, reduce the risk of toxicity, and ultimately facilitate a safer and more flexible treatment.^14^, ^34^ Besides, EVs demonstrate a high potential that their PK properties can be modified by surface structure engineering, although further studies are still required to establish standardised EV structure manipulation methodologies to make them target specific cell types.^73^ Figure 3 demonstrates one of the surface modification methods—PEGylation, which uses PEG to bind anti‐EGFR nanobodies to the surface of the EVs to make them target cancer cells. In the study, the PEGylated EV (EV pi Ega1) with R2‐PEG micelles, which drastically increased EVs binding to EGFR‐overexpressing cells (A431 cells, epidermoid carcinoma cells) compared to cells that lack EGFR expression (NeuroA2 cells).^74^ Another study also suggests that PEGylation of streptavidin on the surface of EVs can improve EVs’ biodistribution by facilitating the conjugation of target components, enabling EVs to target specific cells.^75^ While the engineering of the EV composition enables improving the ability of EVs to deliver drugs or facilitates the uptake and function of drugs through the adjuvant mechanisms, it may also cause unintentional alteration to EVs’ effect on other tissues. Consequently, this can induce undesired effects on untargeted tissues/organs. Therefore, any modification to EVs must be thoroughly considered and account for the content of use.

Since EVs play an essential role in cell communication and can affect the behaviour of recipient cells, it is foreseeable that EVs have the potential to be used as an adjuvant therapy to modulate the cells to enhance or suppress the absorption/metabolism of medications if EVs can be modified with specific signalling structure.^13^, ^31^, ^69^ Even though there are limited studies that apply this theory to clinical trials, many preliminary studies suggested that this is a viable method for future PK modulation. For instance, several studies have shown that 80 different endogenous and xenobiotic enzymes are associated with the presence of EVs. These EVs are involved in local metabolism/detoxification to facilitate the activity of those enzymes, suggesting EVs have the potential to produce similar metabolism‐modulating functions with those enzymes.^76^ Cytochromes P450 (CYP) and UDP‐glucuronosyltransferases (UGT) are enzymes that are essential in the metabolic metabolism of over 50% of drugs; their activities are modulated by the expression of specific genes present in our body; those pharmaco‐genes are also found in EVs.^48^, ^77^ A recent study has found that some plasma EVs contain functional proteins and mRNAs of those enzymes; EVs may be involved in the transportation and activating biogenesis of these enzymes; for instance, it has been discovered that the presence of ethanol can trigger the biogenesis of CYP2E1 and CYP2E1 mRNA containing EVs, leading to accumulation of these EVs in the circulating plasma, which enhances ethanol metabolism. Similarly, this mechanism is also found in the modulation of CYP3A4, which is responsible for the metabolism of several therapeutic medications such as St. Jon's Wort, ritonavir, phenobarbital and many herbal products.^78^ Since many CYP and UGT enzymes play significant roles in the activation or inactivation of medications, and EVs are highly involved in this process, they demonstrate their potential to modulate the PK of different medications.^78^ Studies also demonstrated that EVs could act as a surrogate for drug metabolism and clearance if they are loaded with cargos that can modulate drug metabolising enzymes such as CYP2D6 and CYP3A5, which are rapid metabolisers.^79^, ^80^ With the modulation of these enzymes, we can potentially modulate the metabolism of analgesics (e.g., tramadol), antidepressants (e.g., tricyclic antidepressants), antihypertensives (e.g., metoprolol) and an anti‐cancer agent, tamoxifen.^77^, ^78^ These studies gave us insight into a possible solution for PK/PD‐related problems; if we can establish an effective method to modify the surface or inner structure of EVs, they would have the capability to stimulate/block the transporter/ligand of the selected medication, supply enzyme for local metabolism, modulate gene expression that can produce metabolic enzymes. Hence, EVs have the potential to enhance the PK profile for medications in diseased organs.

**FIGURE 4:**
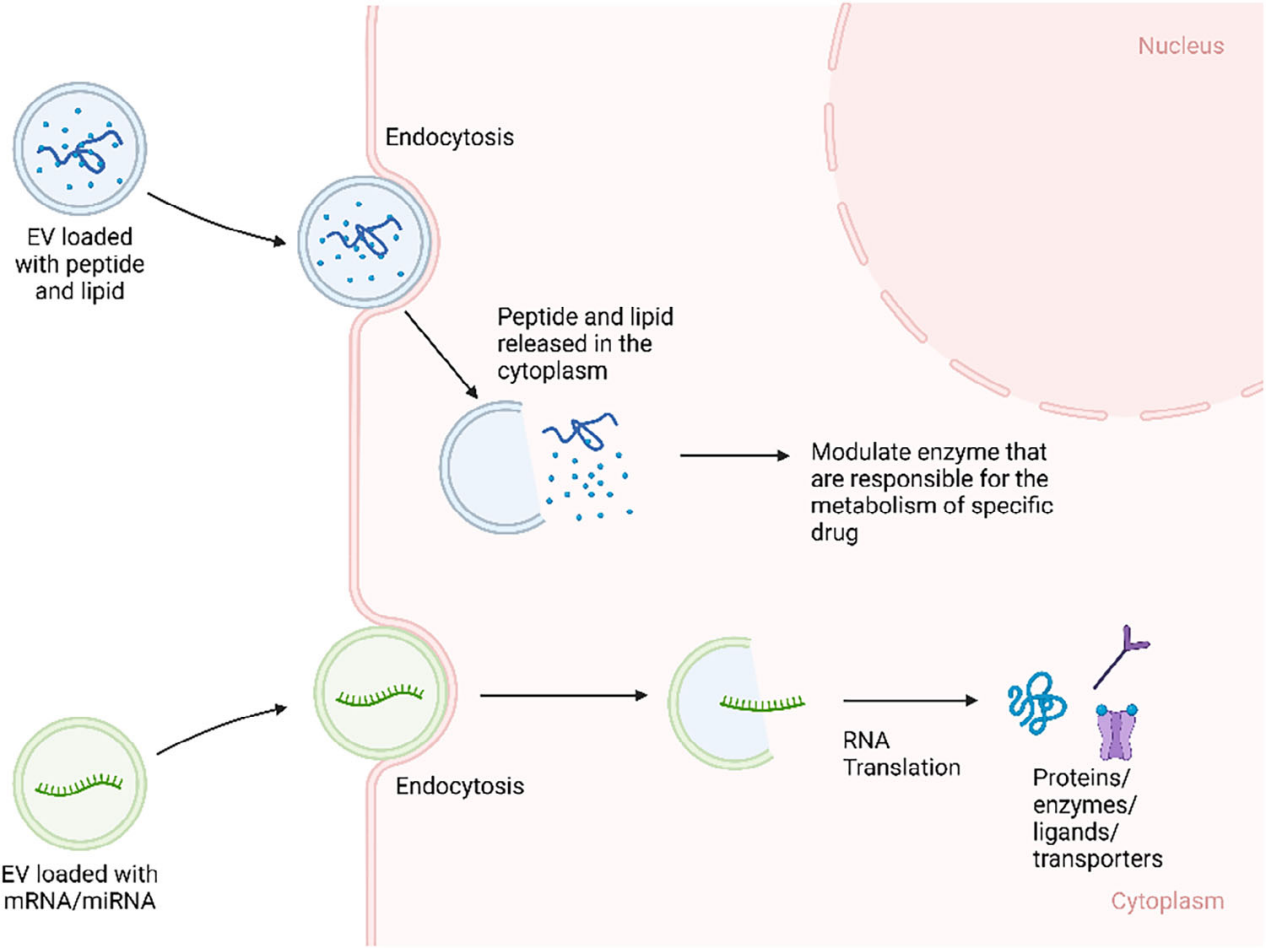
One of the theories of extracellular vesicle (EV) as a modulator of medication's pharmacokinetics (PK). Loading EVs with mRNA or miRNA cargo that can translate and produce proteins with two pathways. (1) Loading EVs with peptides or lipid that can potentially produce signalling effect on the enzymes that are responsible for the metabolism of specific medication, therefore, modulate the metabolism of drugs. (2) Loading EVs with mRNA and miRNA that can directly produce functional proteins/enzymes/ligands/transporters, which promote medication absorption and/or metabolism.

**FIGURE 5:**
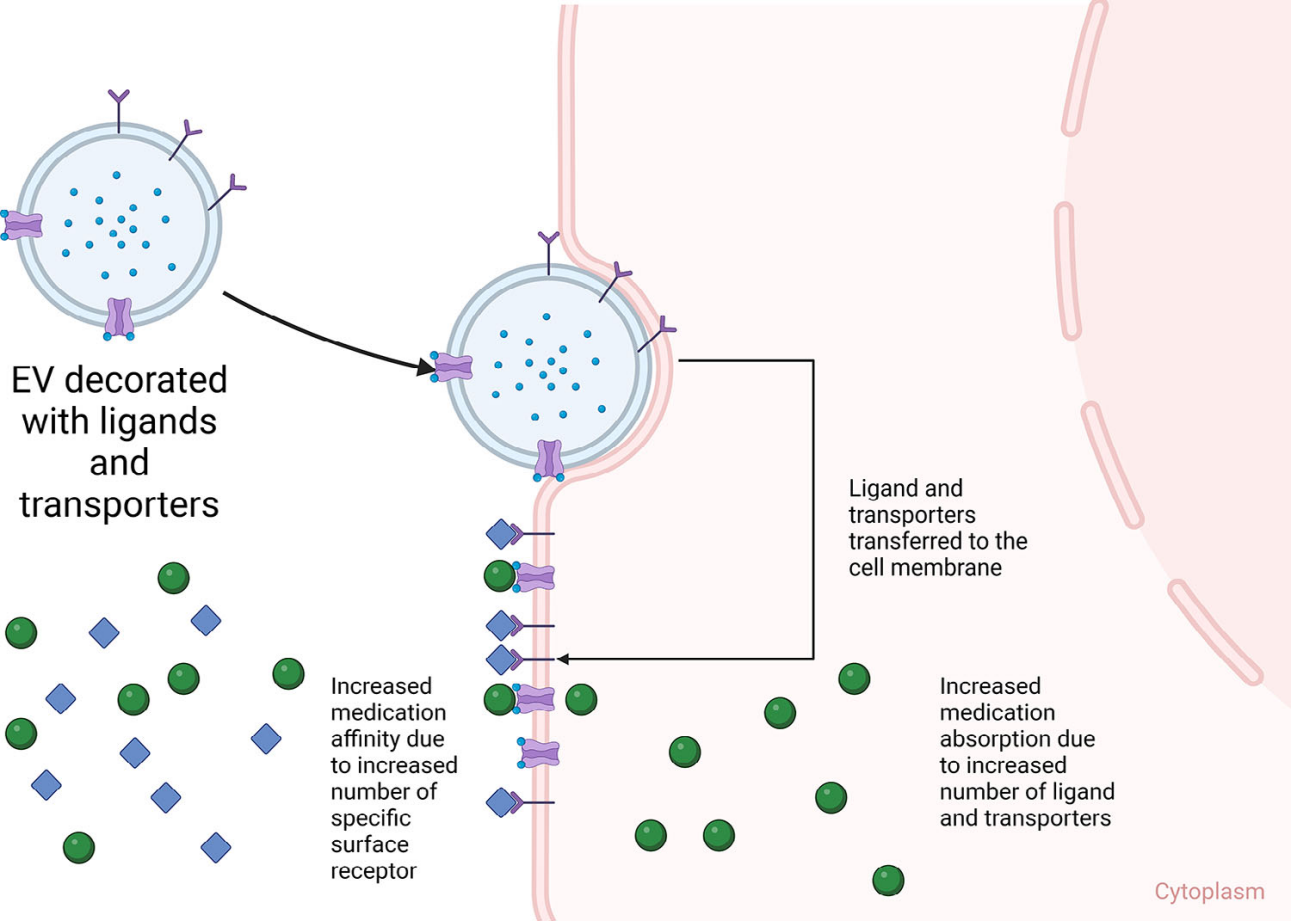
One of the theories of extracellular vesicle (EV) as a modulator of medication's pharmacokinetics (PK). EVs surface engineered with specific ligands and transporters, when they are internalised by the cells via endocytosis, the surface‐engineered ligand and transporters are transferred to the cell membrane, which increases the population of the total ligands and transporters on the cell membrane and, therefore, alters the absorption and/or removal of medications in the cell cytoplasm.

**FIGURE 6:**
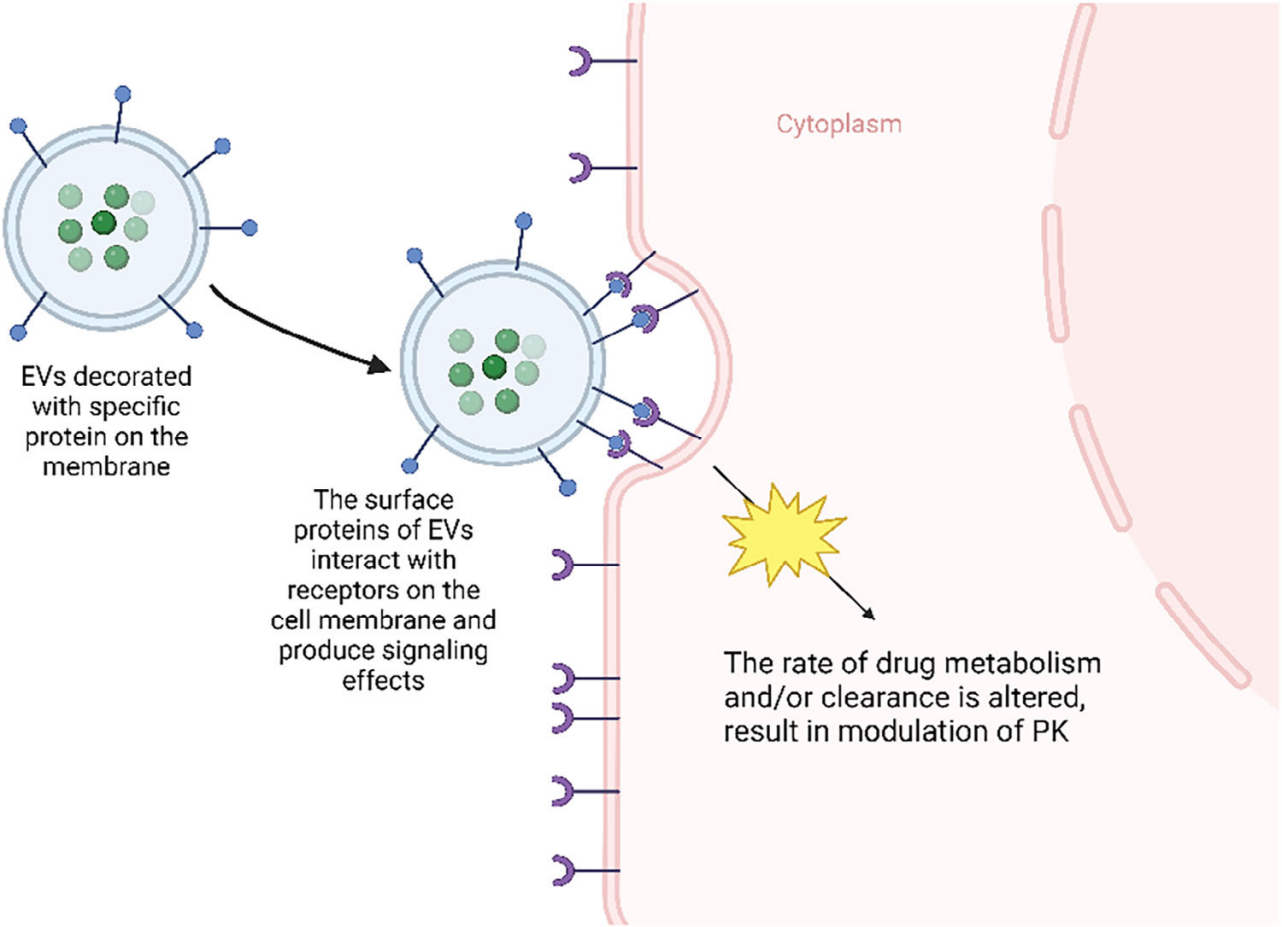
One of the theories as a modulator of medication. Extracellular vesicles (EVs) modified with specific proteins that interact with receptors on the cell membrane to produce signalling effect that can modulate the pharmacokinetics (PK) of drugs.

Thus, EVs are valuable subjects to be studied further as a possible adjuvant therapy that can modulate the PK of medications. Based on these theories, there are several potential methodologies to modify EVs into a modulator of PK. EVs can be loaded with mRNAs and miRNAs that upregulate specific enzyme/protein molecules that are involved in the biotransformation of the medication from its prodrug/pharmacologically inactive form into active drug, resulting in higher active drug concentration in specific cell type/organ/tissue. Since EVs can transport proteins or genetic molecules (mRNAs and miRNAs), particular cargos can be loaded into EVs, such as enzymes that are responsible for the metabolism of specific medications, leading to faster biotransformation from active drugs into inactive metabolites. The miRNA cargo can also up/downregulate the expression of particular genes that are responsible for the synthesis of receptors, which eventually regulate the absorption/action of the medication (Figure 4). EVs can also be surface engineered with specific receptors on the membrane of the EV that can increase the population of receptors on the membrane of recipient cells, which may eventually increase the potency of specific medications that target those receptors (Figure 5). EVs can also be modified with surface proteins that can interact with specific receptors on the cell membrane to exert their therapeutic effects (Figure 6).^81^

### 4.3 EVs as modulators of PD (as adjuvant therapy, pre‐treating/co‐treating)

EVs’ effect on PD of medications is still not sufficiently understood since the quality of EVs as modulators of PD are defined on a case‐by‐case basis, depending on the type of disease, class of medication used, and patients' health condition. However, EVs have shown their intrinsic therapeutic effects, and the effects can be potentially amplified with proper EV engineering. Thus, EVs are considered a safe and foreseeable future adjuvant modulator of PD. A typical example is the addition of immunotherapy in late‐stage melanoma, immunotherapies are added as a co‐treatment to activate CD9 and T cells that enhance the efficiency of chemotherapies eliminating cancer cells. However, introducing a new drug to the therapeutic plan may also cause new drug–drug interactions or introduce new side effects.^82^ EV, with good biocompatibility and their human cell origin, have a lower possibility to trigger adverse reactions, since EV are proven to be effective in stimulating specific immune functions (e.g., using tumour‐derived EV to stimulate T cells) if EV can be surface engineered with a particular set of proteins, they can be used to mimic the function of the immunotherapy and enhance the effectiveness of conventional chemotherapy.^83^ Furthermore, EVs have the ability to change the behaviour of recipient cells. EVs can potentially be used as a pre‐treatment or co‐treatment to set up a cell environment that increases the sensitivity of the diseased cell to the medication and makes healthy cells less responsive to the treatment. In this ideal situation, EVs can produce a protective effect for healthy cells and/or enhance the actions of conventional therapies specifically for diseased cells. Having such an adjuvant therapy can improve the efficacy of the treatment and cause less harm at the same time.

## 5 LIMITATIONS OF CURRENT KNOWLEDGE AND FUTURE STUDY DIRECTIONS

EV shows very promising potential as both drug delivery vehicles and adjuvant therapies for medications that have undesirable PK/PD profiles. However, there are still areas that require more studies and research to gain a comprehensive understanding of the relationships between EV and PK/PD. Many questions regarding the mechanism of EVs’ modulation on PK and PD remain poorly understood, although several studies demonstrated some correlations of EV's modulational effect with their source of biogenesis, intrinsic structure and cargo composition, most mechanism of action are based on speculation during the analysis of preclinical data. Previous studies also demonstrated a lack of effective and scalable EV production and purification methodologies, which pose an obstacle to differentiating the PK and PD of different types of EVs.

On one hand, the diversity of workflows and technologies used in the biomanufacturing and bioprocessing of EVs presents significant opportunities for the biomedical and pharmaceutical industries. However, on the other hand, it introduces challenges related to designing, standardising and regulating EV formulations for clinical applications. For instance, variations in methods for EV isolation and purification result in differences in the subpopulations of EVs obtained and their purity, including co‐isolated media components. These variations influence the downstream physiological effects induced by EVs. Consequently, standardising EVs and their manufacturing protocols becomes challenging.

It is worth noting that isolated EVs typically contain other co‐isolated components, such as cytokines and hormones, which enhance (even work synergistically) or hinder the therapeutic effects of EVs. However, the actual effects of these co‐isolated components are context dependent and might be difficult to establish. This means that depending on the application of EVs the co‐isolates can be beneficial or undesired in the EV preparations. For example, the presence of anti‐inflammatory cytokines is likely to augment the therapeutic effects of EVs in wound healing applications.^7^ While co‐isolated DNAs or their fragments may have deleterious effects and diminish the effects of the genetic cargo of EVs (e.g., miRNA) that is transferred to cells. However, even if the function of these components can be well characterised, the batch‐ and protocol‐dependent heterogeneity of EV preparations raises concerns in the context of reproducibility, safety and efficacy.^84^ Taken together, there is a need to establish precision biomanufacturing and bioprocessing protocols that consider the context of use and will guarantee consistent EV compositions for a given application.

Another challenge for EVs as therapeutics is their accumulation in non‐targeted organs. A significant proportion of EVs tend to accumulate in the liver and spleen, which can impact their therapeutic efficacy because it can scavenge a considerable amount of EVs from circulation before they reach their target organ.^85^ This issue can be mitigated by engineering the surface of EVs that improves the circulation time and biodistribution by coating EVs with albumin binding domains or enhancing targeting by expressing/attaching antibodies on the EV surface.^32^, ^86^ However, the development of EVs’ surface modifications is still in the early stages and adds to the complexity of the regulatory landscape of EVs. Even though many structural engineering processes have been developed, modifying EV structures to meet clinical needs requires further studies and more comprehensive preclinical and clinical trials.^87^

In addition, there are limited studies that demonstrate a dose‐dependent response to EVs. Currently, there is no standardised guideline for the determination of EV dosage. One of the reasons could be the batch‐to‐batch variability of EV preparations,^7^ which makes it difficult to establish the correlation between EVs’ dosage and their clinical therapeutic effects.

Storage and stability are other challenges for EVs to be utilised as therapeutics. EVs demonstrate significant degradation at room temperature. Storage at −20°C or −80°C can provide better stability, however, repeated freeze‒thaw may lead to EV aggregation and decreased functionality.^88^ Freezing also requires specialised media with cryoprotectants and stabilisers to prevent EV damage. The choice of storage buffer can additionally have a significant role in EV stability. Commonly used buffer solutions (e.g., phosphate buffer solution) may cause aggregation in long‐term storage (more than 26 weeks).^89^, ^90^ Although there are specialised buffers (e.g., phosphate buffer solution supplemented with human albumin and trehalose that are proven to improve the stability during storage, however), their use may require the buffer exchange steps before the application.^89^

While one can identify general challenges for EV applications across various fields, it is critical to emphasise that the significance of these challenges depends on the context of use. For instance, when we use EVs for downstream applications such as intravenous injections, topical applications, ex vivo stimuli for cell activation, or as active ingredients in cosmetic and veterinary formulations, each application will have distinct regulatory requirements, even though they have not been fully established yet.

Additionally, most existing studies are wild‐type animal studies, and a comprehensive database of EVs’ toxicity, immunogenicity, therapeutic effects and PK/PD profile is still not established for the human body since there is a lack of clinical studies, which constrain the selection of EVs in clinical studies. Furthermore, EVs’ surface modification/structure engineering is still at early stages. Even though many structural engineering processes have been discovered, modifying EVs’ structures to fulfill desirable properties and gain certain therapeutic functionalities is still challenging and the structure modification technology requires further studies and more comprehensive preclinical and clinical trials (Table 3).

**TABLE 3:** Context of use‐dependent challenges and opportunities for extracellular vesicles’ (EVs’) applications.

Barriers to developing EV formulation	Current solution	Limitations
Multiple and non‐standardised EV isolation and purification methods	Compliance with characterisation guidelines for reproducibility and reporting of EVs (EV‐TRACK platform); minimal information for studies of EVs^91^	Limited considerations of the context of use that should guide the requirements for the EV preparation composition and purity Method‐dependent ‘purity’ of isolated EVs (isolation method and protocol define the composition of EV preparations) Laborious and expensive characterisation, quality control and validation methods
Accumulation in non‐targeted organs	EV surface engineering	The EV surface/structure modification technologies require further studies and more comprehensive preclinical and clinical trials EV engineering processes add to the complexity of the regulatory procedures
Limited data in dosage management	Animal studies and clinical trials Multiorgan‐on‐a‐chip studies	Batch‐to‐batch variability Animal studies may not fully reflect EVs’ behaviour in the human body
EV stability, storage and delivery	Use of specialised buffers for storage at lower temperatures (−20°C or −80°C) Lyophilisation Hybridisation with lipids	A limited number of buffers are available, and some are still under development Freezing and thawing of EVs may lead to aggregation and unpredictable loss of EV functionality A need for buffer exchange Unknown loss of the efficacy/potency of EVs after resuspension into the final, application‐dependent buffer EV formulations require further studies

## AUTHOR CONTRIBUTIONS

*Conceptualisation, writing—original draft, writing—review and editing and visualisation*: Jiaqi Liu. *Writing—original draft and writing—review and editing*: Joel Z. Nordin. *Writing—review and editing*: Andrew J. McLachlan. *Supervision, conceptualisation, writing—original draft, writing—review and editing and visualisation*: Wojciech Chrzanowski. All authors reviewed the results and approved the final version of the manuscript.

## CONFLICT OF INTEREST STATEMENT

Ass. Prof. Joel Z. Nordin has stock interest and is a consultant for Evox Therapeutics.

## FUNDING INFORMATION

This research did not receive any specific grant from funding agencies in the public, commercial or not‐for‐profit sectors.

## ETHICS STATEMENT

Not applicable since no clinical trials were conducted as part of this review manuscript.

## Data Availability

Not applicable.
